# Challenges and support for quality of life of youths living with HIV/AIDS in schools and larger community in East Africa: a systematic review

**DOI:** 10.1186/s13643-019-0980-1

**Published:** 2019-02-26

**Authors:** Emmanuel Kimera, Sofie Vindevogel, Jessica De Maeyer, Didier Reynaert, Anne-Mie Engelen, Fred Nuwaha, John Rubaihayo, Johan Bilsen

**Affiliations:** 1 Department of Public Health, School of Health Sciences, Mountain of the Moon University, Fort Portal, Uganda; 2 Department of Orthopedagogy, Health and Social Work, Faculty of Education, University College Gent, Gent, Belgium; 3 Health and Social Work, Faculty of Education, University College Gent, Gent, Belgium; 4 Department of Occupational therapy, Health and Social Work, Faculty of Education, University College Gent, Gent, Belgium; 5 School of Public Health, College of Health Sciences, Makerere University, Kampala, Uganda; 6 Department of Public Health, Mental Health and Wellbeing research group, Vrije Universiteit Brussels, Brussels, Belgium

**Keywords:** HIV/AIDS, Youth, Quality of life, Systematic review, Psychosocial support, East Africa, Challenges, Living with HIV

## Abstract

**Background:**

Youths living with HIV/AIDS (YLWHA) experience innumerable challenges within schools and the larger community. Nonetheless, these environments are potential sources of support for such youths. This review provides a synthesis of evidence about these challenges and support available for YLWHA to inform the design and implementation of interventions that support the wellbeing of youths living with HIV/AIDS in an East African context.

**Methods:**

We searched MEDLINE, Cumulative Index to Nursing and Allied Health Literature (CINAHL), Web of Science, and Cochrane central registry of systematic reviews and randomized control trials for studies conducted in East Africa and published in English in the last 10 years (March 2007 to March 2017). We also searched Google Scholar and reference lists of all included studies. We purposed to include both qualitative and quantitative data but no quantitative data merited inclusion. We analyzed qualitative data using a framework thematic analysis.

**Results:**

We included 16 primary studies conducted in clinic and community settings that used qualitative or mixed methods. Three overarching themes—psychosocial wellbeing, treatment and health, and disclosure of HIV status together with a sub-theme of stigma that was important across the three overarching themes—were the basis for analysis. In each overarching theme, a duality of challenges versus support was reported. Psychosocial wellbeing included subthemes of challenges in schools and larger community, financial challenges, domestic violence, sexual and reproductive health challenges, and psychosocial support.

**Conclusion:**

YLWHA experience numerous challenges and support needs, some of which occur in schools and affect their QoL. The effects of these challenges are poor health and educational outcomes as well as school dropout. The schools in which youths spend most of their formative years have not provided adequate support for YLWHA. This review identified that although most of the challenges that studies identified arose from within schools and that a few supportive approaches were available, none of the studies explored how these supportive approaches would work in schools. It was additionally identified that stigma complicates challenges of living with HIV/AIDS necessitating interventions for the wellbeing of YLWHA to understand and address HIV-stigma and its ramifications. Such interventions ought to be sustainable in schools, culturally appropriate, and multidisciplinary in order to promote the general health of all students.

**Electronic supplementary material:**

The online version of this article (10.1186/s13643-019-0980-1) contains supplementary material, which is available to authorized users.

## Background

Globally, over 36.7 million people were living with the human immunodeficiency virus and acquired immune deficiency syndrome (HIV/AIDS) in 2015 [1]. Around 2100 youths are infected with HIV every day [2], the majority of whom are living in low- and middle-income countries, with 85% in sub-Saharan Africa. The growing cohort of perinatally infected youths before prevention of mother-to-child transmission (PMTCT) programs together with the high infection rate during adolescence accounts for the high prevalence among youths [3]. Cognizant of the fact that these countries have youthful populations, HIV/AIDS forms a major wicked problem that continues to require due attention in this region.

The increased availability of antiretroviral treatment (ART) has drastically reduced mortalities and morbidities in infected youths over the last decade [4–6]. Perinatally infected children are now more likely to grow into adulthood and beyond, also in resource-limited countries [7]. These medical advances have been transforming HIV/AIDS from a death sentence to a chronic condition [7–10]. As a chronic condition, HIV/AIDS requires long-term attention and a shift from cure to care and support [11] with emphasis not exclusively on prolonging life but also on increasing quality of life (QoL) [12].

The Quality of Life framework is imperative in supporting people living with a chronic condition since it focuses on general wellbeing and satisfaction with life in different domains that may be affected by the condition [13]. Furthermore, it moves beyond a deficit-oriented approach by placing people and their perceptions, goals, values, qualities, and strength at the center to enhance a life of good quality [14]. By approaching wellbeing as a multidimensional construct and studying it from the subjective experiences of people, it has proven to offer a thorough understanding of the real impact of a chronic condition on multiple life domains among various populations living with long-term support needs [15–18]. Additionally, QoL framework puts emphasis on personal context by understanding people in the settings that are significant to them [13]. Like all chronic conditions, HIV/AIDS affects innumerable aspects of daily living and imposes numerous challenges upon the lives of youths and their surroundings [19–22]. Youths living with HIV/AIDS (YLWHA) are more likely to experience emotional, behavioral, and cognitive difficulties due to both the direct neuropsychiatric effect of the HIV/AIDS infection on their brain functioning and the indirect effects related to a wide range of stressors, including the complexities of adolescence and experienced stigma that compound living with HIV/AIDS [19, 20, 23–26]. Research has further demonstrated that HIV/AIDS has a strong negative impact on individuals’ social integration, as their status often evokes stigmatization and discrimination in their community leading to poor social support [2, 25, 27]. YLWHA are also more prone to experiencing difficulties with independent functioning, school participation, and educational performance [22, 23, 26].

Experienced challenges and supportive measures mostly come into effect in natural living environments. Since youth spend most of their time within schools, the school community is a pivotal natural environment for cognitive, personality, emotional, behavioral, social, and physical development [28]. For YLWHA, the school conditions may either facilitate or hinder their QoL, among other things through affecting their self-care behavior and adherence to ART and other interventions [29]. It has further been noted that many youths struggle with disclosure and stigma in the school community [29]. According to the Human Rights Watch [30], the neglect of millions of YLWHA is rapidly fueling school dropout across East and Southern Africa. In this region, HIV/AIDS is singled out as a major factor driving up to about 50% of all cases of school dropouts [31]. Living with HIV/AIDS in school communities may be complicated considerably when this environment fails to respond resiliently to disclosure and budding special needs. In fact, it may jeopardize these youths’ QoL.

To our knowledge, no documented systematic literature review of challenges and support among YLWHA in (school) communities has been undertaken in East Africa to synthesize this vital evidence and to inform the design and implementation of interventions tailor-made for the East African populace and context. This could relate to why most HIV/AIDS-related interventions for youth in this region such as the Presidential Initiative on AIDS Strategy for Communication to Youths (PIASCY) programs in Uganda and other abstinence, being faithful, and condom use (ABC) strategies in the whole region have a preventive agenda and do not cater for the QoL of those youths already infected with HIV/AIDS. Subsequently, this review has been undertaken to pool evidence from independent studies carried out in East Africa, to have a broader, substantiated, and contextually sensitive perspective on challenges and support available for YLWHA. The specific objectives of the review are twofold: (1) to review evidence on challenges that YLWHA experience in schools and larger community and (2) to review evidence on support available and interventions programed for YLWHA in schools and the larger community. This novel review will form a basis for prospective studies, policies, and interventions aimed at minimizing challenges and enhancing support for YLWHA in schools and in the community at large in order to facilitate their QoL and the attainment of the United Nations’ sustainable development goal 3 (Ensure healthy lives and promote well-being for all at all ages) and goal 4 (Ensure inclusive and equitable quality education and promote lifelong learning opportunities for all) [32] in East Africa.

## Methods

We conducted a qualitative review of studies that involved qualitative methods of data collection such as FGDs, interviews, role-play, and photovoice following the guidance of the Cochrane handbook of systematic reviews [33] and the Preferred Reporting Items for Systematic reviews and Meta-Analyses (PRISMA) statement of 2009 [34]. We appreciated the need to register our review protocol with PROSPERO to avoid duplication and bias when conducting the review but we did not undertake this since we did not meet the second inclusion criterion of PROSPERO that requires the review to have at least “one outcome directly related to human health”. Although our results directly relate to human health, they are not results of measures or tests and therefore do not qualify as outcomes. However, we searched PROSPERO and did not find any ongoing or complete review similar to ours.

### Criteria for selecting studies for this review

#### Types of studies

We planned to include studies that employed quantitative, qualitative, and mixed methods designs for both data collection and analysis, but only qualitative and mixed methods studies were retained.

#### Target participants

Youth (12–19 years) living with HIV/AIDS were targeted for this review although studies in the general population were considered if data on YLWHA was reported and if these youths constituted over 50% of the participants in such studies. This age category of youth was selected because in East African education systems, such youths are expected to be attending primary or secondary school.

#### Phenomenon of interest

The included studies reported on at least one of the following phenomena of interest to this review: *challenges* faced by YLWHA or *support* available in schools or communities to enhance the wellbeing of YLWHA. A challenge was considered to be any factor whose presence or absence has the propensity to negatively affect the well-being of YLWHA. These could operate at the individual level, community level, or policy level. We considered support as any form of assistance available to YLWHA to address their unique challenges and needs. An individual’s ability to cope was considered as a form of self-support while community and institutional support was also explored. We included support initiatives as interventions aimed at improving the QoL of YLWHA other than standard care and treatment provided by health workers.

### Search methods

We formulated one comprehensive search strategy with stringent inclusion and exclusion criteria. We used the Cochrane HIV/AIDS group collaboration search strategy [33] AND search strings of youth OR similar AND challenges OR similar OR support OR similar AND schools OR community OR similar (see Additional file 1 for the search strategy used in Cochrane database). The schools in which YLWHA spend most of their time were considered a special environment and therefore included in the search strategy, but communities outside school were also considered. We searched the following electronic databases for eligible scientific articles: CINAHL through host EBSCO, searched in May 2017 to provide references from the nursing field; MEDLINE through PubMed, searched in March 2017 to provide reference from the field of public health; Cochrane central registry of controlled trials and systematic reviews, searched in April 2017 to provide randomized control trials and reviews conducted in the field of HIV/AIDS; Web of science, searched in March 2017 to provide multidisciplinary references; and Google scholar, searched in May 2017 to provide additional possible references. In each database, monthly email alerts were created using the email address of one author (EK) for updates of new studies that conformed to our search strategies. By the time of revision of this manuscript, no new study merited inclusion. Reference lists of all included studies were also searched for additional studies using the same inclusion and exclusion criteria.

### Inclusion/exclusion criteria

We included studies published in English in the last 10 years (1997–2017) and conducted in East Africa (Uganda, Kenya, Tanzania, Rwanda, Burundi, and South Sudan). We included this time restriction in our search in order to get the most recent evidence given that research on HIV/AIDS has expanded exponentially over the past decades. Additionally, access to ART in East Africa has increased in the last decade [35]. This points to increased survival of children with HIV and emergence of challenges and support needs related to leaving with HIV as a chronic condition. Studies exploring these challenges and support needs would therefore be expected within this time frame. We also restricted our search to East Africa because these countries have more or less similar economic and cultural contexts. The history of how they have dealt with HIV is also different from that of other areas like South Africa or West Africa, and therefore, sub-Saharan Africa would be too broad. We also realized that due to the extensive research in HIV in South Africa, our findings and conclusions based on the entire Sub-Saharan region would be skewed more towards South Africa. We excluded narrative reviews, studies in which participants were not clearly defined and those in the general population if data on youth (12–19 years) was not explicitly reported.

### Data extraction and management

All search results were imported into a citation management software Zotero Standalone, and duplicates were removed. Guided by a set of inclusion criteria, EK scanned through all titles and abstracts and excluded articles that clearly had irrelevant titles. Abstracts and keywords of the remaining articles were then scrutinized after which the full texts of all retained articles were retrieved. These were then assessed by EK in consultation with SV to establish their merit for inclusion. EK and SV collaborated closely and resolved any doubts that arose about studies, and consensus was achieved on all studies that were included or excluded. Data extraction from included studies was done using a pre-tested data extraction form (Additional file 2) and evolved through an iterative process guided by the purpose of the review. Two review authors, EK and SV, independently extracted the data after which they converged and compared the extracted data. In cases of discrepancies in the extracted data, the original articles in which those discrepancies arose were revisited and such discrepancies were resolved by discussion to arrive at a consensus. The extracted information included study details (citation and location), participant details (number, age, gender), methods (data collection and analysis methods), and findings. We did not encounter any case of missing data in the included studies.

### Assessment of risk of bias in studies

We did not obtain quantitative studies to include in the review, and for the included mixed methods studies, quantitative data were excluded because they did not focus on our phenomenon of interest. For that reason, we did not perform a risk of bias assessment on the included studies since it is not appropriate for qualitative studies [36].

### Assessment of the quality of included studies

Critical appraisal of qualitative research is widely debated, and currently no consensus exists on methods, tools, and whether it should even be done [37, 38]. In many studies, such appraisal has not led to exclusion of papers from the synthesis [39–41]. Sandelowski and Barroso [42] also noted that critical appraisal was more of an exercise of judging a written report and not the research process itself. This kind of appraisal also tends to favor papers published in qualitative oriented journals that allow lengthy papers that enable authors to elaborate on the research process [43, 44]. On the backdrop of this, we did not conduct a critical appraisal but judged included studies to be of high quality because they were published papers in peer-reviewed journals.

### Data analysis

In accordance with recommendations by Cochrane qualitative review methods group, we analyzed qualitative data reported in the results section of included studies using a framework thematic analysis approach [45]. Quantitative data in mixed methods studies were excluded from the analysis because they were not targeted findings for this review. In the framework approach, the thematic analysis was guided by an analytical framework. This enabled us to explore data in depth while simultaneously maintaining an effective and transparent audit trail, thereby enhancing the rigor of the analytical process [46]. During the first of the five stages called *familiarization*, we repeatedly read thoroughly the included studies with the objectives of the review in mind. In the second stage of *identifying a thematic framework*, an analytical framework was developed based on the objectives of the review and inductive preliminary coding of data in three articles. Alterations to the analytical framework were discussed between EK and SV to accommodate other themes that emerged as the coding proceeded. The overarching themes thus included were psychosocial wellbeing, treatment and health, and disclosure of HIV status together with a sub-theme of stigma that was important across the three overarching themes. In the third stage of *indexing*, we re-read the selected studies paying keen attention to their data and framework themes. The existing themes in the included studies were deconstructed and reconstructed in line with the framework themes. The fourth stage of *charting* involved rearranging data codes according to framework themes to which they related in order to form charts. Within each theme, related categories were grouped to form subthemes in an iterative way as the data abstraction and charting process went on. Appropriate quotes of perspectives of participants in the primary studies were also included in the charts within categories they belonged. The abstraction and synthesis focused more on the perspectives of the respondents in the included studies rather than the interpretations of the authors of the primary studies. During the last stage of *mapping and interpretation*, we defined the concepts and found associations between the framework themes, and subthemes and synthesized the findings.

## Results

### Search results

**Fig. 1:**
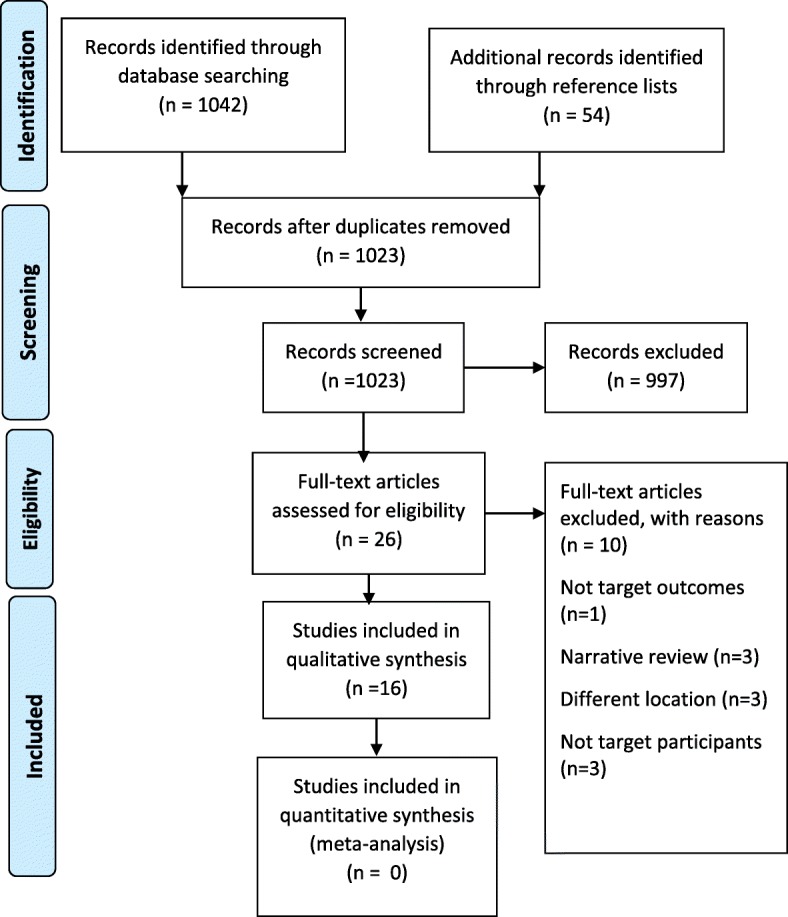
Study flow diagram

We got a total of 1096 titles and abstracts and retained 26 eligible articles. After assessing full texts of the 26 articles, 10 were excluded because they had either different findings (*n* = 1) or different participants (*n* = 3) or different location (*n* = 3) basing on our inclusion and exclusion criteria or were narrative reviews (*n* = 3). We finally included 16 studies in the qualitative synthesis [47–62] and none in the meta-analysis. The study flow diagram (Fig. 1) shows the study selection procedure.

### Description of included studies

#### Settings

All studies included in this review were conducted in East African countries. Seven of these [47–53] were conducted in Uganda. Four studies [54–57] were done in Kenya. From Tanzania, four studies were included and these are [58–61]. One study [62] was from Rwanda. Eleven of the studies [48–55, 57, 58, 62] were carried out at health facilities or research centers, 4 of them [47, 56, 60, 61] were carried out in communities while 1 study from Kenya [54] was conducted in both health facility and community. Health facility-based studies were conducted at health centers providing HIV treatment or both HIV treatment and HIV research. Two of the community-based studies [60, 61] included participants involved in home-based care (HBC).

#### Study designs

All the 16 studies included in this review employed qualitative designs. Two studies, one from Tanzania [59] and another from Uganda [53], used mixed methods combining both qualitative and quantitative methods. The qualitative methods used were focus group discussions (FGDs), key informant interviews (KII), in-depth interviews (IDI), photovoice (PV), and role play (RP). Many studies used these methods in combination. FGDs were used by 9 studies, IDI by 9, and KI by 3 while PV and RP were each used in 1 study. The quantitative designs used were cross-sectional [59] and records review [53].

#### Study participants

In almost all the included study, authors sought the perspectives of YLWHA themselves although many studies also included primary caregivers, health care providers, and teachers as respondents. For purposes of this review, we considered youth from 12 to19 years of age. We therefore scrutinized the age categories of YLWHA in studies to ensure that at least 50% of them fell in our target age group. In one Ugandan study [52] and one Kenyan study [55], YLWHA were not involved. These two studies sought the perceptions of health care providers and expert clients who assist with adherence support to YLWHA.

#### Interventions

In two of the included studies, authors qualitatively evaluated an intervention. One study from Tanzanian [61] sought the perceptions of YLWHA about home-based care (HBC) in order to improve it to meet the needs of YLWHA. In a study from Uganda [50], the authors sought youths’ perspectives on the acceptability and feasibility of a Short Message Service (SMS)-based intervention named Reminding Adolescents To Adhere (RATA) aimed at promoting adherence to treatment among HIV-positive youths. The results of the evaluation of these two interventions are presented in the “Findings” section of this review below.

### Characteristics of included studies

#### Findings (Table 1)

**Table 1:** Characteristics of included studies

Study/location/settings	Participants	Study design	Data analysis methods	Aim	Subthemes	Findings
[47] Fournier (2014) Uganda/community	13 HIV-infected and orphaned youths (5 females) 12–18 years, living in a group home	Photovoice and FGD	Thematic analysis	To explore the experience of orphaned, HIV-seropositive children who live in a group home in Semi-urban Western Uganda.	Hopes and dreams Material resources Social support Stigma and discrimination Psychological, emotional and social challenges	Needs, social support, and challenges
[48] Mutumba (2015) Uganda/clinical research center	38 HIV-positive youths (20 females) 12–19 years	Interviews	Thematic analysis	To identify the psychosocial challenges and coping strategies among perinatal HIV-infected adolescents in Uganda	HIV stigma Disclosure Adherence Coping strategies	Challenges of living with HIV and coping strategies in youths
[49] Bakeera-kitaka (2008) Uganda/health facility	75 HIV-infected youths (35 females) 11–21 years	FGDs	Thematic analysis	To assess sexual and reproductive health needs and problems as well as determinants of sexual risk taking among young people living with HIV aged 11–21 years attending the pediatric infectious disease clinic in Kampala.	Information and misconceptions about sexual and reproductive health	Barriers to adopting protective behavior, behavioral skills adopted by youths for protective behavior, health care providers’ perception on sexual reproductive health-related needs of YLWHA
					Adolescents’ motivations for adopting protective behaviors	
					Perceived barriers for adopting protective behaviors	
					Behavioral skills adopted by adolescents for protective behaviors	
					Health care providers’ perceptions on SRH-related needs of YPLH	
[50] Rana (2015) Uganda/health facility	^a^39 HIV-infected youths 14–24 years	FGDs	Thematic analysis	To explore perspectives of youth on the acceptability and feasibility of SMS-based interventions.	Feasibility of the Intervention	Perceived challenges of the intervention and suggestions for improvement.
					Programmatic Challenges and Suggestions	
					Pathway Mechanisms	
[51] Kawuma (2014) Uganda/medical research centers	26 HIV-positive youths (12 females) 11–13 years, 10 Caregivers, 5 Health workers.	Interviews	Thematic analysis	To examine the reasons for non-adherence to ART among children and why they may not report when they miss their treatment.	Not knowing the reasons why, one should take the drugs	Reasons for nonadherence, reasons for not disclosing nonadherence.
					Lack of food and side-effects	
					Fear of being seen by others	
					Lack of time	
					To protect and maintain relations with carers and healthcare workers	
					Fear of being scolded	
[52] Inzaule (2016) Uganda/health facility	11 nurses, 9 adherence counselors, 5 medical doctors, 5 expert patients, 3 pharmacists.	Interviews and FGDs	Thematic analysis	To assess challenges to long-term adherence in adolescents and adults in three regional HIV-treatment centers in Uganda.	Unstructured treatment holidays	Challenges to adherence disaggregated for youths and adults
					Delays in disclosing HIV status to perinatally infected children	
					Diminishing or lack of family support	
					Perceived and experienced stigma in boarding schools	
					Declining or lack of clinic support	
					Temporary migrants and challenges with treatment access	
					Disclosure in intimate relationships	
					Treatment-related factors	
					Staff shortages and missed counseling opportunities	
[53] Nabukeera-Barungi (2015) Uganda/health facilities	^a^336 HIV-infected youths 10–19 years, 46 Caregivers	^b^Mixed methods: qualitative (interviews, key informant interviews and FGDs) and quantitative retrospective record review.	Thematic analysis	To describe the level and factors associated with adherence to antiretroviral therapy as well as the 1 year retention in care among adolescents in 10 representative disctricts in Uganda.	Barriers to adherence to ART	Level of adherence, factors associated with adherence and retention in care
					Facilitators of adherence and retention in care	
[54] Abubakar (2016) Kenya/medical research center	12 HIV-infected youths (3 females) aged 12–17 years and 7 HIV-uninfected youths (5 females) 12–17 years, Caregivers of HIV-infected youths (*n* = 11), Community health workers (*n* = 8), Teachers and education administrators (*n* = 6)	Interviews	Thematic analysis	To investigate psychosocial challenges faced by HIV infected adolescents on the Kenyan coast.	Poverty as a salient challenge for families with HIV	Psychosocial challenges
					Poor mental and physical health	
					Confronting a school system that is not responsive to their needs	
					Partial disclosure to family and peers	
					Stigma	
					Medical adherence	
[55] Hagey (2015) Kenya/health facility	40 health care providers	Interviews	Thematic analysis	To explore barriers and facilitators adolescent females living with HIV face in accessing contraceptive services.	Stigma of sexual promiscuity in accessing contraception without a partner	Barriers to access contraception and facilitators to contraception
					Concerns of negative parental attitudes towards adolescent sexual activity	
					Discouragement from seeking contraceptive services due to being different from peers	
					Provider interactions and bias of adolescent sexual activity influence contraceptive services offered	
					Targeted youth-friendly services encourage adolescents to seek contraceptive services	
					Ease of accessing contraception through integration of HIV and contraceptive services	
[56] Lypen (2015) Kenya/community	53 HIV-infected youths (26 females) 18–27 years.	FGD	Thematic analysis	Tobetter understand the complex support system among HIV-positive youth and related coping mechanisms	Types of social support	Social support types, sources and their influence on management and coping with HIV
					Sources of social support	
[57] Gachanja (2015) Kenya/health facility	7 HIV-infected youths (3 females) and 5 HIV- negative youths (3 females) 12–17 years.	Interviews	Thematic analysis	To explore post-disclosure experiences of children.	Acceptance of illness	Challenges following disclosure and coping mechanisms
					Stigma and discrimination	
					Medication consumption	
					Sexual awareness	
					Coping mechanisms	
[58] Ramaiya (2016) Tanzania/health facility	24 HIV-infected youths (18 females) 13–23 years.	Interviews	Thematic analysis	To identify salient psychosocial and mental health challenges of HIV-positive youth in a resource-poor Tanzanian setting	Living with HIV	Psychosocial challenges of living with HIV
					Domestic and Family Environments	
[59] Nyogea (2015) Tanzania/health facility and community	116 HIV-infected youths (49 females) 2–19 years for the quantitative part, ^a^35 HIV-infected youths 13–17 years for qualitative part, 21 Caretakers and 2 Health workers	^b^Mixed methods: quantitative cross-sectional and qualitative FGDs and Interviews.	Thematic analysis	To estimate adherence levels and find the determinants, facilitators and barriers to ART adherence among children and teenagers in rural Tanzania.	Facilitators of treatment adherence	Adherence levels, determinants of adherence, barriers and facilitators of adherence. Disclosure of HIV status
					Treatment adherence barriers	
[60] Busza (2013) Tanzania/community	14 HIV perinatally infected youths (5 females) 15–19 years, 10 Caregivers and 12 home-based care providers	Interviews	Thematic analysis	To explore how adolescents in Tanzania with HIV experience their nascent sexuality, as part of an evaluation of a home-based care program	Postponing sexuality	Perceptions of youths living with HIV about sexuality and HIV
					Sex, risk, and health	
					Expectations for the future	
[61] Busza (2014) Tanzania/community	14 HIV perinatally infected youths (5 females) 15–19 years, 10 Caregivers and 12 home-based care providers	FGDs and interviews	Thematic analysis	To examine the experiences of adolescents living with HIV in Tanzania in order to improve home-based care to better meet their needs	Adolescents’ participation in care	Perceptions of youths and HBC providers about home-based care
					Perceptions of current services	
					HBC providers’ experiences	
[62] Mutwa (2013) Rwanda/health facility	42 HIV-positive youths (19 females) 12–21 years, 10 Caregivers	Interviews, FGDs and role-playing	Thematic analysis	To understand adherence barriers for Rwandan adolescents	Desire to be Healthy	Challenges of living with HIV and social support
					Stigma and Desire for Privacy	
					Disclosure of HIV Status	
					Acceptance, Isolation and Depression	
					Social Support and Living Situations	
					Medication and Regimen Issues	

*FGD* focus group discussions

^a^Participant not disaggregated by sex

^b^Quantitative results not reported since they were not targeted findings of this review

**Fig. 2:**
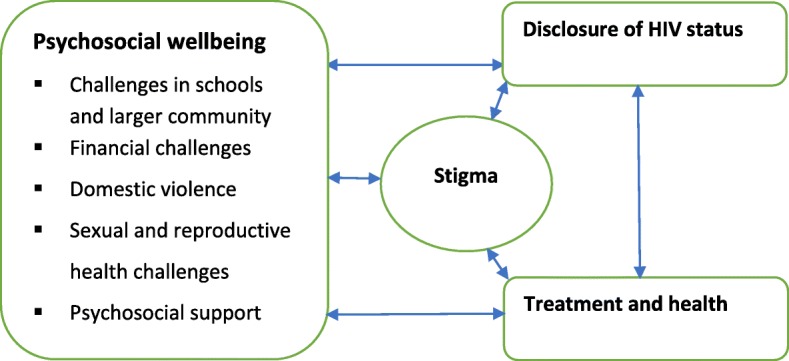
Study themes

We identified three main interrelated themes intermediated by a subtheme of stigma. Stigma linked to challenges identified across the themes of psychosocial wellbeing, disclosure of HIV status and treatment and health as shown in Fig. 2. Due to the interrelatedness of the main themes, the findings in one main theme occasionally blended with those of another.

### Psychosocial wellbeing of YLWHA

#### Challenges in schools and larger community

This was reported in half of the included studies [48, 50, 52, 54, 57–59, 62]. In many of these studies, the school was deemed a challenging environment for YLWHA. It was found that YLWHA tend to feel unique from their peers due to their HIV status but also due to the medication they have to take recurrently to survive leading to internalized stigma [62]. The school was reported to be a highly stigmatizing environment given the insensitive and bullying attitude of some students. It was additionally noted that some teachers, HIV un-infected youths, and caregivers had potentially stigmatizing views, opinions, or beliefs about HIV/AIDS [54, 57]. One of such was that youth contracted the disease by participating in prostitution. In extended families, discrimination from stepmothers/fathers, aunts, uncles, or other children was depicted in separation of plates, utensils, and shower basins [48]. Unequal treatment of the children also occurred for instance in enrolling YLWHA in inferior schools compared to those in which siblings without HIV enrolled and not being allowed to play with non-HIV infected siblings [48]. Unequal distribution of financial and emotional support was also reported [58]. Combining HIV-related activities and school was also a challenge for many YLWHA. Attending medical treatment often made them lag behind in education, as they had to miss a day of school every month to attend the clinic [54]. Also, in the SMS-based intervention [50], prohibited phone use at school was a key hinderance to youth’s adherence.

The lack of adequate support from health workers, parents, and extended family members were identified as drivers for non-adherence [50, 59]. Many YLWHA were lost to follow-up when they reached 18 years in the transition to the adult clinic because of failure to integrate with adult-patients, reduced attention from health workers, and long waiting hours [52]. The shortage of staff in health facilities leading to heavy workloads resulted in inadequate counseling for YLWHA and failure to address their needs [52]. In some cases, YLWHA did not get the desired attention when they were counseled in a group with adults [59]. It was additionally reported that YLWHA got more support to stay on medication from their biological parents than caretakers [59]. The perinatally infected were troubled by how and why they got HIV and expressed blame and anger towards their parents for infecting them [57].

… I came to counseling and the counselor taught me how to take medicine and the consequences … I used to cry then after some time maybe like two months that is when I started accepting myself. Now I feel just like a normal human being, I just take it like a cold … But I still blame my dad coz he knew he was positive yet he let my mother give birth to me and my mother never knew she had the disease (girl) ([57], p7).

#### Domestic violence

One study [58] reported on instances of domestic violence towards YLWHA. It was noted that on several occasions and particularly in case of perinatal infection, YLWHA had lost one or both parents. This left them with no choice but to be taken care of by relatives (uncles and aunts) and in the worst scenario to take care of themselves. In the caretaker families, YLWHA often experienced physical and emotional violence. They were insulted, battered, discriminated, and referred to as “walking dead.” For those that lost their biological mothers, they had to bear the mistreatment from a stepmother. These violent acts drove YLWHA out of schools and homes to streets where they got involved in criminal acts.

#### Financial challenges

Four studies [51, 53, 54, 58] highlighted financial stress/poverty as a challenge affecting YLWHA and as one of the causes for non-adherence to treatment [51, 53]. Perinatally infected youth were particularly prone to financial challenges due to the risk of having lost one or both parents who would have fended for them or having parents too sick and weak to engage in income-generating activities. Those living with caretakers also experienced financial constraints due to the additional burden to the caretaker families. As a result, YLWHA could not attend medical appointments and collect their ART due to lack of transport fares [53, 58]. A number of respondents mentioned difficulties enrolling and continuing in school as well as getting proper meals and clothing due to such financial deficiencies [54, 58].

I lack school fees, food and fare … to come here [to the health clinic] (13 years old) ([54], p2)

#### Sexual and reproductive health challenges

Challenges related to SRH were reported in 4 studies [49, 55, 57, 60]. The youths in the age target for this review [12–19 years] are characterized by a strong attraction to the opposite sex and a high desire to engage in sex. For YLWHA, reconciling their HIV status with maintaining confidentiality and having an intimate relationship is a quandary. In one study [49], YLWHA revealed some misconceptions about sexual and reproductive health (SRH) which are detrimental to their own health and that of their sexual partners. One such misconception was from a 16-year-old boy who thought that if he got overawed by sexual desires, he could have sex with a girl and ask an adult to remove sperms [49]. Peer pressure to engage in sexual activity was reported by some youths [57], and they had a challenge of overcoming such pressures in the bid to conform to their peers and avoid being suspected of having HIV. Getting into a relationship, however, also seemed a big predicament. They wondered if they would be accepted by their partners because of their status and feared to transmit HIV to others [60].

I have not thought about it [relationships/marriage] yet … you might be afraid of infecting the person you live with. Otherwise, if you tell someone he may reject you … so you count yourself useless. It is better to stay single (female, 15 Dar es Salaam) ([60], p91–92).

Some YLWHA desired to be like their friends who were already involved in sexual relationships, and they wanted to have sex at least once before they died since they did not believe they would live long to reach the right age for sex [49]. It was further reported that some female HIV-positive youths engaged in sex despite their knowledge of consequences of unprotected sex in exchange for money because they were poor and vulnerable to manipulation by men [49]. Some reported use of alcohol and watching pornographic movies with their peers [60], which caused them to lose their sense of judgment and to engage in risky sexual acts. Parents and caretakers were also uncertain regarding the appropriateness and timing for introducing SRH education. Mothers worried about causing additional emotional distress to their children on top of HIV if they talked about sex, and others held views that people with HIV should never engage in sex [60].

Challenges faced by female YLWHA to access contraceptive services were also highlighted. Contraceptives would enable them to prevent unwanted pregnancies and potential vertical transmission of HIV [55]. They feared that attending the health facility for contraceptives would imply to the community that they were sexually active at a young age. In their perceptions, this would attract community stigma since, in many African cultures, sex is a taboo for adolescent youths and the unmarried [55]. Moreover, many adolescent youths are unmarried and seeking contraceptives would seem to other people that they are promiscuous/prostitutes [55]. This stigmatization was a drawback to contraceptive access for YLWHA.

The challenge is … they still have no partner. They are not like a couple. So, for them to freely come and say that “me I’m practicing sex” is still an issue (male nursing officer, health center) ([55], p3).

Those seeking care with caregivers feared to ask for contraceptives since this would attract questions from their caregivers about their sexual activity. To mitigate this, youth friendly services were suggested as reported under psychosocial support. Providers also doubted which contraceptive method to advance among YLWHA. Many suggested abstinence or condom use while hormonal methods were not promoted [55].

#### Psychosocial support

Nine studies [47–49, 55–58, 60, 62], reported on various forms of psychosocial support available to YLWHA. The Kenyan study [56] identified that newly diagnosed youths relied mostly on emotional support that involves comfort, empathy, or consultation; information support to help them adjust to their diagnosis by giving them advice, factual knowledge or suggestions; appraisal support which involves giving them constructive feedback, assurance or validation and instrumental support which involves giving youth something tangible like a service, physical object or tangible skill [56]. Sources of psychosocial support for YLWHA identified were family, friends, clinicians, counselors, support groups, religion, and partners [48]. Peer support for YLWHA throughout their lives was prominently reported [47, 57, 58].

If you don’t have friends, you feel lonely and you cannot be happy at all … you walk as if you are not walking. But if you have someone, you feel strong and in case you have any problem, that friend can help you ([47], p5).

HIV counselors and clinical staff were handy in providing HIV education and life skills for positive living. Youth also relied on spirituality and religion as a source of strength and support to live with HIV [58, 62]. The hopes and dreams of YLWHA were also seen as a form of inner drive to live positively. Youth expressed desires to be successful in life and to be respected in their communities. This motivation strengthened them and enabled them to confront HIV challenges with a perspective of a good future [47].

After getting money, I will build a house. Then start my own business. I want to be self-employed. I will be the happiest person in the world ([47], p5).

The YLWHA reported receiving material support in form of food, school fees, and drinks from different individuals and organization [47]. These compassionate acts strengthened them.

As a way of supporting SRH of YLWHA, youth-friendly services were offered in many health facilities whereby youth-specific days and services were designated to specifically cater for the needs of YLWHA. This allowed them to access the health facilities without the worry of meeting adults who would judge them for engaging in premarital sex [55]. It was reported that YLWHA required adequate space and time to discuss contraception, free contraceptive services, integration of contraception with HIV services, and adequate contraception counseling. One provider noted:

The moment they arrive here, we have a friendly language, we receive them with positive attitude … we educate them and give them services for free (female nursing officer, sub-county hospital) ([55], p5)

Four studies [47–49, 57] explored ways in which YLWHA coped with psychosocial challenges of living with HIV/AIDS. Youth reported using a variety of strategies that enabled them to avoid worrying about HIV and death. Some engaged in activities that distracted their thoughts in order to try and forget about being HIV-positive. These included getting busy with academic work or home chores and chatting with friends [48]. Playing games such as football [47, 48], watching TV, and listening to music [48, 57] as well as praying to God to give them strength [57] were other distractors. Non-disclosure or disclosure to few trusted persons as well as lying about frequent illnesses and daily medication were employed to cope with potential stigma and its consequences such as loss of friendship, discrimination, and gossip [48]. To cope with SRH challenges, it was reported that many avoided environments that could cause them to think about sex such as romantic situations or bad peer groups. Some refused any sexual activity and discouraged advances on grounds of religion or by disclosing their HIV status to scare those pursuing them. The use of condoms to avoid unwanted pregnancies and the spread of HIV was also reported by older YLWHA, albeit inconsistent use. A few youths stated masturbation as a way of easing the desires to engage in sex with a partner, but this was unacceptable to most of them [49].

#### Home-based care (HBC) intervention

The HBC program provides a wider consortium of psychosocial services to support treatment initiation, adherence, HIV counseling, and testing for partners and family members as well as long-term psychosocial care within homes [60]. The providers are usually voluntarily working, well-respected older women from the communities often also living with HIV. As reported by [60], every YLWHA was linked to a HBC provider who periodically visited the YLWHA to follow up on clinic appointments, provide support with treatment adherence as well as any other psychosocial support and practical guidance. The HBC provider also interacted with other family members and sensitized them about health including HIV. Participating YLWHA valued the offered hope, confidence, and special attention. They also appreciated the practical assistance for referrals to other organizations or help in arranging medical appointments. Some participants cited scenarios where HBC providers used their own resources to buy food and medicine, although this was not part of the HBC program. The providers also evaluated the program positively and, though working voluntarily or for a small facilitation, enjoyed serving youth.

### Disclosure

#### Challenges

Nine studies [47, 48, 50, 51, 53, 57, 58, 61, 62] reported on challenges related to disclosure of HIV status. The fear of gossiping, ridiculing, teasing, and losing of friendship caused YLWHA to conceal their status from some family members, teachers, and peers which again led to isolation and depression [48, 58, 62]. They were found to use several ways to conceal their HIV status, like covert medication use, shunning other identified HIV-positive peers and not sharing their family history [58]. Disclosure to partners would lead to rejection or acceptance or extreme anger, and due to this unpredictability, some YLWHA feared to get involved in romantic relationships [58].

With my relatives, it hasn’t affected anything but with other people it has. For example, I can’t be in any intimate relationship with anyone because I don’t know his status and I just can’t tell every guy who wants to be with me that I am HIV positive. I need time and courage to do that (female respondent) ([58], p7)

For those who chose to conceal their status in a romantic relationships, taking medication was a challenge and they did not adhere to their treatment [53]. Unintentional disclosure often arose in for instance boarding schools due to lack of privacy in dormitories and lack of confidentiality among some school staff [48]. To avoid this, YLWHA refrained from taking medication in the presence of their peers, avoided noisy medicine bottles, and avoided frequenting the school clinic [48]. Delayed disclosure of HIV status by parents or caretakers to perinatally HIV-infected youths was also a barrier to adherence. Some children reported rejecting drugs because they did not know why they had to take them [51]. For some, the repeated illnesses and visits to clinics caused them to suspect that they had HIV infection and they were anxious for someone to tell them. When this did not happen, out of frustration, they deliberately stopped taking drugs as a way of compelling caretakers to disclose to them [51]. When disclosed to, many YLWHA experienced shock and struggled with the knowledge that they were HIV-positive [57, 58]. The self-denial that usually accompanied disclosure caused depression and isolation in many, which in turn prevented them from taking their medicine [63]. They were always preoccupied with fear that they would soon die [47].

The fear of disclosure was reported as a key limitation in the RATA SMS-based intervention [42] and in the Home-based care (HBC) intervention [61]. The sharing and deliberate checking of phones by other people risked revealing the status through the messages sent from the clinics [50]. It was also reported that youth feared that HBC providers would disclose their status in the community [61].

I would like the providers to keep the secrets of their clients … These service providers have a tendency of broadcasting their client’s problems [HIV/AIDS] that I don’t like at all. They tell and tell. I don’t like it. There should be some confidentiality (girl,) ([61], p139).

#### Disclosure support

None of the included studies reported on support concerning the theme of disclosure.

### Treatment and health

#### Challenges

Living with HIV/AIDS requires lifelong treatment with antiretroviral drugs. Medication-related stressors emerged as common themes in half (*n* = 8) of the included studies [48, 51–54, 57, 59, 62]. In these studies, respondents mentioned several inhibitors of treatment adherence. As reported earlier, the fear of stigma and unintentional disclosure augmented medication challenges [48]. Intentional missing of drugs [57], postponing doses, or even throwing away medicine [54] was reported.

Recently he mentioned … . Grandma, I am tired of all these medicines and I do not feel happy. I told him you will have to continue taking anyway, what else can we do? (Grandmother caregiver) ([54], p5).

The school environment played a major role in medication adherence. The lack of privacy in boarding schools [53], segregation by teachers and other students [59, 62], and stigma in schools [54, 62] were moreover reported as causes of non-adherence to treatment in schools.

When I was going to school, they gave me drugs for three months and I kept them in my bag. As each one had his/her own bed, I had to cover myself with the sheet to swallow the tablets … however, someone tried to steal my stuff by cutting open my bag … when they found my medicine and scattered it on my bed. When other students came back, they asked to whom the drugs belonged and I said I didn’t know. So, you can understand that keeping medicine in dorm is risky (Focus Group) ([62], p4)

Being playful like their peers also caused YLWHA to forget taking their drugs [51, 59]. The desire to experience a drug-free life like HIV-negative peers, drug fatigue, and pill burden [52], as well as depression and misconceptions about treatment [53], were other causes of non-adherence to treatment. Most YLWHA start taking drugs when they are very young and later on their commitment to drugs reduces because their health improves and they see no need to continue taking them [53]. Often times when youth saw some health improvements [59] or when they learned that ARVs do not cure HIV [53], they became reluctant to continue with treatment. Drug fatigue also resulted from side effects of the drugs [51, 52]. Recurrent infections like skin rashes due to immunosuppression additionally affected the health of YLWHA [54].

Some grown-up children may sometimes cheat that they have taken drugs while they have not, because they are tired and the parents believe that they have already taken the drugs (FGD, female) ([59], p10).

#### Treatment support

Six studies [48, 50, 53, 58, 59, 62] reported on support to promote adherence to medication. YLWHA appreciated the availability and effectiveness of lifelong ART [58] and its capacity to cause them to feel better and reduce infections [59, 62].

Ooh, the treatment is good, like me I wasn’t like this, before taking this treatment. I had rashes all over my body and I was so thin until my fellow children were avoiding me and said I had AIDS, but when I started taking the medicine, my condition changed and I am now big and healthy [Focus Group Discussion] ([59], p5).

Information about treatment adherence and continuous encouragement from peers, counselors, and health workers enabled YLWHA to adhere to their treatment. Scheduling of clinic visits during school holiday, provision of food and transport to the clinic, short waiting time, and telephone calls and text messages from the clinic were also identified as other ways YLWHA were supported to adhere to treatment [53]. It was reported further that treatment gave some of the YLWHA hope, that one time a cure will be discovered and they would become HIV free [53]. Youth in day schools relied on reminders such as phone alarms and prompts from parents/caregivers to take their medicine. In some instances, reminders from teachers and significant others were utilized. In boarding schools, YLWHA set discrete alarms, some carried pills in their school bags, and some kept medication at the school clinic while some sought permission from school authorities to return to the dormitories during class time in order to take their medicine [48]. Some YLWHA managed to integrate taking medication into their daily activities so that it became part of their daily routine [48].

##### The RATA/SMS-based intervention

One intervention to improve treatment adherence codenamed RATA (Reminding Adolescents To Adhere) involved use of mobile phone Short Message System (SMS) to remind adolescents to take their medicine. Short text messages reminding adolescents to take their drugs were sent from clinics to mobile phones of YLWHA or their caretakers in order to improve treatment adherence and social support through encouraging messages [50]. Majority of the YLWHA felt that the RATA/SMS intervention helped them to improve their adherence. It nevertheless had major limitations related to owning and sharing of mobile phones and the restricted use of phones at school and at home as already reported under psychosocial wellbeing.

## Discussion

The aim of this review was to synthesize evidence on challenges and support for quality of life of YLWHA within schools and larger communities in East Africa. While we envisioned to include qualitative, quantitative and mixed methods studies, only qualitative and mixed method studies were retained. We also intended to vividly explore the school community but none of the included studies were conducted in schools, though data relating to the school environment was reported. Most of the studies were health facility-based and few conducted in the community. We intended to include support initiatives as interventions aimed at improving the QoL of YLWHA other than standard care and treatment by health workers. Our search identified only 2 such studies out of 16 included studies. One of these two was aimed at improving adherence to medication while the other was aimed at providing a wide range of psychosocial support services. Both were evaluated qualitatively by seeking opinions of beneficiaries [YLWHA] and providers. This shows a deficit of evidence-based interventions to address HIV/AIDS challenges and provide support to youth within East Africa, especially in school communities.

Reported challenges and supportive strategies were categorized into three interrelated main themes; psychosocial wellbeing, disclosure of HIV status, and treatment and health, intermediated by a subtheme of stigma. Despite medical advances and their facilitation of longevity, living with HIV remains challenging and being young appears to aggravate this. The multifaceted challenges identified in this review were also found in earlier reviews [63, 64]. In the review covering HIV/AIDS knowledge in sub-Saharan Africa [63], youth were found to experience challenges relating to their physical health, emotions, and schooling as those found in this review. The review on interventions to reduce HIV/AIDS stigma [64] showed that HIV-stigma has a huge bearing on both disclosure of status and adherence to treatment as also reported in this review and other primary studies [65–68]. In order to promote the wellbeing of YLWHA, stigma should be understood and addressed at peer, family, and community levels since it links directly to disclosure, treatment adherence, and schooling as evidenced in this review. Peer-led stigma can be reduced by HIV awareness programs in schools and individual-level interventions that teach HIV-infected children how to seek support and openly talk to trusted peers [69, 70]. Such interventions must foster an enabling environment for open communication and build the skills of key adults in the lives of YLWHA such as caretakers, teachers, and health care providers [67]. They should also hinge on multiple disciplines to be relevant in addressing the multifaceted challenges.

In the reviewed studies, the school was portrayed as a very challenging environment for YLWHA. It appeared to be a key driver for non-disclosure [67] and non-adherence [5, 67, 71–74], relating to the high levels of stigma and discrimination within the school environment [66]. Despite these challenges, schools are environments with potential to be a strong source of support needed to foster resilience. YLWHA may receive additional psychosocial support from teachers and peers in school if stigma is overcome and limited disclosure is encouraged [67]. Training of school staff and management is crucial to increase access to psychosocial support for YLWHA. Since YLWHA are challenged with finances as seen in the reviewed studies, the education system should address barriers to schooling such as fees and food [75, 76]. Intersectoral collaborations guided by new policies should also be explored to ensure medical supplies for YLWHA within schools to address adherence, retention in care, and absenteeism from school. This, however, requires focused training for school healthcare workers. A multidisciplinary approach to school interventions similar to the Wellness Academics and You (WAY) intervention in the USA [77] should be encouraged as the framework to address not only HIV issues but all those that affect youth.

With regard to psychosocial support, the main sources of support were family, religion, peers, clinicians, and partners. The school authorities and staff did not feature as sources of support despite their role in the upbringing of youth. Implied resources to facilitate living with HIV/AIDS were mostly related to provision of correct and timely information related to HIV/AIDS and SRH. In the studies reviewed, it was evident that matters of SRH of YLWHA were deliberately ignored by parents/caretakers and health workers. Sexual abstinence was encouraged but given that youth typically have a high urge for sex and high propensity to succumb to peer pressure, YLWHA need more support on how to be in a healthy sexual relationship as well as on how to use contraceptives. Given that this review revealed extant challenges and support related to SRH at large, information needs regarding HIV/AIDS would better be framed and addressed within a larger context of SRH that does not only target YLWHA but all youth. Interventions should also be grafted in the legal and cultural features of the region. Moreover, interventions aimed at supporting youth in living with HIV/AIDS could draw hugely on the strategies already employed and deemed efficient by the youth themselves. Throughout the review, YLWHA expressed ways in which they coped with identified challenges and support needs in order to live positively. The review identified coping strategies which included seeking distraction, practicing religion, self-motivation, and rationalization.

Review of evidence for interventions aimed at supporting YLWHA in East Africa showed potential benefits from the RATA-SMS and HBC interventions. The effectiveness of SMS interventions has been assessed in several reviews and primary studies [78–85]. Despite variations in settings and participants, all studies reported that SMS reminders significantly improved adherence except one study from Cameroon [83], involving adult participants. The potential to promote adherence by use of SMS reminders notwithstanding concerns about the use of SMS observed in this review are similar to those observed by [85] regarding confidentiality, sharing of phones, and restricted phone use in schools. Besides these concerns, one can be skeptical about the sustainability of the SMS intervention in resource-limited settings. Knowing that many YLWHA reported financial challenges in this review, owning a mobile phone could be unfeasible and would appear a luxury amidst their financial woes.

Regarding the second intervention, this review supported by results from other studies shows high feasibility of the HBC intervention to offer psychosocial support to YLWHA as well as promoting ART adherence especially for YLWHA [86, 87] and HIV/AIDS population in general [88]. These studies, however, did not pay specific attention to unique characteristics of youth. There is a need for more research on this intervention and for more evidence-based interventions for this population in general. One of the questions to be addressed is how the HBC program would involve YLWHA in boarding schools. In the qualitative systematic review of global perspectives of interventions to improve adherence, Ma et al. [89] identified similar barriers to the HBC programs as in our review.

Although both RATA and HBC interventions had potential to improve the wellbeing of YLWHA, their key limitations show that program designers should pay keen attention to socio-economic contexts of the end users (YLWHA) and implementers (health workers) as depicted in the suggestions for improvement. Alternative sustainable interventions within schools where youth spend most of their time should be explored. These recommendations could facilitate future design of sustainable, culturally appropriate, and multidisciplinary interventions in school communities in East Africa since these are non-existent in schools as evidenced in this review.

### Quality of evidence, strength, and limitations of this review

We restricted our search to studies published in English. In addition, we focused on scientific peer-reviewed studies and did not include grey literature such as studies and interventions from non-government organizations. This might have introduced a publication bias in our study. However, the comprehensive search strategy and the variety of databases used enabled us to obtain all possible studies for inclusion. The two interventional studies included in this review were evaluated qualitatively. We cannot therefore base on this evaluation to ascertain their effectiveness. Larger randomized control trials are needed. None of the included studies used a long-term ethnographic approach in which observations would be made, as they unfold in a natural environment. The results are based on perceptions provided by YLWHA, caretakers, and health care providers whose responses may be greatly influenced by their perceptions of possible benefits. The perceptions of the program designers involved in the RATA SMS intervention and HBC program were not sought in the included studies. These would be vital in understanding their willingness to address some of the identified limitations in the programs and our recommendations for extending/adjusting them to the school communities. We did not search EMBASE database which also indexes biomedical journals and articles. This could have led to a possible omission of some studies for inclusion in the review. Given that the research on HIV/AIDS is rapidly evolving, continuous attention should be paid to newly emerging evidence that could inform the support of quality of life for YLWHA.

## Conclusion

The YLWHA experience numerous challenges and support needs that affect their QoL, but there is lack of evidence-based interventions to address HIV/AIDS challenges and to provide support to such youth in East Africa especially in school communities. This review showed that although most of the challenges that studies identified arose from within schools and that a few supportive approaches were available, none of the studies explored how supportive approaches would work in schools. The identified challenges cut across different domains of wellbeing for YLWHA and can better be addressed by sustainable culturally appropriate multidisciplinary interventions.

## Additional files

Additional file 1: Search strategy. (DOCX 12 kb)

Additional file 2: Data extraction form format. (DOCX 12 kb)

## Abbreviations

ABC: Abstinence, being faithful, and condom use
AIDS: Acquired immune deficiency syndrome
ART: Antiretroviral therapy
ARVS: Antiretrovirals
FGD: Focus group discussion
HBC: Home-based care
HIV: Human immunodeficiency virus
PIASCY: Presidential Initiative on AIDS Strategy for Communication to Youth
PMTCT: Prevention of mother-to-child transmission
QoL: Quality of life
RATA: Remind Adolescents to Adhere
SMSy: Short Messaging System
SRH: Sex and reproductive health
WAY: Wellness, Academics and You
YLWHA: Youth Living With HIV/AIDS
