# Supplementary material for: Challenges and support for quality of life of youths living with HIV/AIDS in schools and larger community in East Africa: a systematic review
Source: Syst Rev. 2019 Feb 26;8:64. doi: 10.1186/s13643-019-0980-1 (PMC6390353; doi:10.1186/s13643-019-0980-1)
Supplement: Supplementary file 1 — Search strategy. (DOCX 12 kb) [file 13643_2019_980_MOESM1_ESM.docx]

**Additional file 1: Search strategy used in Cochrane database**

ID Search

#1 MeSH descriptor: [Adolescent] explode all trees

#2 "youth":ti,ab,kw (Word variations have been searched)

#3 "Child":ti,ab,kw (Word variations have been searched)

#4 children:ti,ab,kw (Word variations have been searched)

#5 MeSH descriptor: [Adolescent] explode all trees

#6 "teenager":ti,ab,kw (Word variations have been searched)

#7 MeSH descriptor: [Young Adult] this term only

#8 MeSH descriptor: [Adolescent] this term only

#9 #1 or #2 or #3 or #4 or #5 or #6 or #8

#10 MeSH descriptor: [HIV] this term only

#11 HIV:ti,ab,kw (Word variations have been searched)

#12 "human immune deficiency virus":ti,ab,kw (Word variations have been searched)

#13 MeSH descriptor: [HIV] this term only

#14 MeSH descriptor: [Acquired Immunodeficiency Syndrome] this term only

#15 hivaids:ti,ab,kw (Word variations have been searched)

#16 #10 or #11 or #12 or #13 or #14 or #15

#17 challenges:ti,ab,kw (Word variations have been searched)

#18 schools:ti,ab,kw (Word variations have been searched)

#19 "communities":ti,ab,kw (Word variations have been searched)

#20 "intervention":ti,ab,kw (Word variations have been searched)

#21 support:ti,ab,kw (Word variations have been searched)

#22 support initiative:ti,ab,kw (Word variations have been searched)

#23 "psychosocial support service":ti,ab,kw (Word variations have been searched)

#24 "counseling":ti,ab,kw (Word variations have been searched)

#25 "counselling":ti,ab,kw (Word variations have been searched)

#26 support programs:ti,ab,kw (Word variations have been searched)

#27 "supportive care":ti,ab,kw (Word variations have been searched)

#28 support needs:ti,ab,kw (Word variations have been searched)

#29 coping strategy:ti,ab,kw (Word variations have been searched)

#30 #18 or #19 or #20 or #21 or #22 or #23 or #24 or #25 or #26 or #27 or #28 or #29

#31 MeSH descriptor: [Quality of Life] this term only

#32 health related quality of life:ti,ab,kw (Word variations have been searched)

#33 value of life:ti,ab,kw (Word variations have been searched)

#34 "well-being":ti,ab,kw (Word variations have been searched)

#35 experiences:ti,ab,kw (Word variations have been searched)

#36 #31 or #32 or #33 or #34 or #35

#37 #9 and #16 and #30 and #36
