# Supplementary material for: Challenges and support for quality of life of youths living with HIV/AIDS in schools and larger community in East Africa: a systematic review
Source: Syst Rev. 2019 Feb 26;8:64. doi: 10.1186/s13643-019-0980-1 (PMC6390353; doi:10.1186/s13643-019-0980-1)
Supplement: Supplementary file 2 — Data extraction form format. (DOCX 12 kb) [file 13643_2019_980_MOESM2_ESM.docx]

**Additional file 2: Data extraction form format**

| Date of extraction |  |
| --- | --- |
| Full citation |  |
| Purpose of the study |  |
| Study design |  |
| Participants (sample) |  |
| Intervention |  |
| Exposure |  |
| Outcomes |  |
| Note |  |
